# Language Barriers in Organismal Biology: What Can Journals Do Better?

**DOI:** 10.1093/iob/obad003

**Published:** 2023-01-28

**Authors:** B Nolde-Lopez, J Bundus, H Arenas-Castro, D Román, S Chowdhury, T Amano, V Berdejo-Espinola, S M Wadgymar

**Affiliations:** Biology Department, Davidson College, Davidson, NC 28035, USA; Department of Integrative Biology, University of Wisconsin–Madison, Madison, WI 53706, USA; School of Biological Sciences, University of Queensland, St Lucia, QLD 4072, Australia; Department of Curriculum & Instruction, School of Education, University of Wisconsin–Madison, Madison, WI 53705, USA; School of Biological Sciences, University of Queensland, St Lucia, QLD 4072, Australia; Institute of Biodiversity, Friedrich Schiller University Jena, Dornburger Straße 159, 07743 Jena, Germany; Helmholtz Centre for Environmental Research - UFZ, Department of Ecosystem Services, Permoserstr. 15, 04318 Leipzig, Germany; German Centre for Integrative Biodiversity Research (iDiv) Halle-Jena-Leipzig, Puschstr. 4, 04103 Leipzig, Germany; School of Biological Sciences, University of Queensland, St Lucia, QLD 4072, Australia; School of Biological Sciences, University of Queensland, St Lucia, QLD 4072, Australia; Biology Department, Davidson College, Davidson, NC 28035, USA

## Abstract

In the field of organismal biology, as in much of academia, there is a strong incentive to publish in internationally recognized, highly regarded, English-language journals to promote career advancement. This expectation has created a linguistic hegemony in scientific publishing, whereby scholars for whom English is an additional language face additional barriers to achieving the same scientific recognition as scholars who speak English as a first language. Here, we surveyed the author guidelines of 230 journals in organismal biology with impact factors of 1.5 or greater for linguistically inclusive and equitable practices and policies. We looked for efforts that reflect first steps toward reducing barriers to publication for authors globally, including the presence of statements that encouraged submissions from authors of diverse nationalities and backgrounds, policies regarding manuscript rejection based on perceived inadequacies of the English language, the existence of bias-conscious reviewer practices, whether translation and editing resources or services are available, allowance for non-English abstracts, summaries, or translations, and whether journals offer license options that would permit authors (or other scholars) to translate their work and publish it elsewhere. We also directly contacted a subset of journals to verify whether the information on their author guidelines page accurately reflects their policies and the accommodations they would make. We reveal that journals and publishers have made little progress toward beginning to recognize or reduce language barriers. Counter to our predictions, journals associated with scientific societies did not appear to have more inclusive policies compared to non-society journals. Many policies lacked transparency and clarity, which can generate uncertainty, result in avoidable manuscript rejections, and necessitate additional time and effort from both prospective authors and journal editors. We highlight examples of equitable policies and summarize actions that journals can take to begin to alleviate barriers to scientific publishing.

## Abstract (es)

Resumen

En el campo de la biología organísmica, al igual que en el mundo académico en general, existe un gran incentivo para publicar en revistas científicas de lengua inglesa que son reconocidas internacionalmente y que poseen gran prestigio con el fin de avanzar profesionalmente. Esta expectativa ha creado una hegemonía lingüística en la publicación científica en la que los académicos para quienes el inglés es una lengua adicional se enfrentan a barreras adicionales para lograr el mismo reconocimiento científico que los académicos que hablan inglés como primera lengua. En este estudio examinamos las instrucciones para autores de 230 revistas de biología organísmica con Factor de Impacto igual o superior a 1.5 en busca de prácticas y políticas lingüísticamente inclusivas y equitativas. Buscamos iniciativas que reflejen pasos iniciales hacia la reducción de barreras de publicación para autores a nivel mundial. Estas incluyen la presencia de anuncios que incentiven el envío de trabajos por autores de diversas nacionalidades, políticas relacionadas al rechazo de manuscritos debido a la percepción de insuficiencias en el inglés, prácticas de revisión conscientes de prejuicios, disponibilidad de recursos o servicios de traducción y edición, la publicación de resúmenes o traducciones en idiomas adicionales al inglés y la disponibilidad de licencias que permitan a los autores (u otros académicos) traducir su trabajo y publicarlo en otro lugar. También contactamos directamente a un subconjunto de revistas para comprobar si la información que aparece en las instrucciones para autores refleja con exactitud sus políticas y los ajustes que harían. Comprobamos que las revistas y los editores han avanzado poco en el reconocimiento o reducción de barreras lingüísticas y en la promoción de igualdad lingüística. Al contrario de nuestras predicciones, las revistas asociadas a sociedades científicas no parecen tener políticas más inclusivas en comparación con las revistas que no pertenecen a ninguna sociedad. Muchas políticas carecen de transparencia y claridad, lo que puede generar incertidumbre, dar lugar a rechazos evitables de manuscritos y exigir tiempo y esfuerzo adicionales tanto a los futuros autores como a los editores de las revistas. También destacamos ejemplos de políticas equitativas y resumimos las medidas que las revistas pueden adoptar para empezar a aliviar los obstáculos de publicación científica.

## Abstract (zh)

概要

生物学の分野では、他の多くの学術分野と同様、国際的に認知され高い評価を得ている英文学術誌で論文を発表し、キャリアアップを図ろうという強い動機付けが存在する。このため、英語が母語でない研究者は、英語を母語とする研究者と同等の評価を得るために、さらなる障壁に直面するという問題が生じている。本研究では、インパクトファクターが1.5以上の生物学分野の雑誌230誌を対象に、言語的にどれだけ包括的で公平な方針をもっているか、著者ガイドラインを調査することで明らかにした。多様な国籍や背景を持つ著者からの投稿を奨励する記述の有無、英語力の不足を原因とする原稿のリジェクトに関する方針、偏見に配慮した査読方針、翻訳·・編集リソースやサービスの有無、英語以外の要旨・や本文の許容、著者 (または他の研究者) が自分の原稿を翻訳して別途出版できるようなライセンス条項を雑誌に設けているかなど、論文出版に対する障壁を減らすための第一歩となる取り組みに着目した。また、一部の雑誌には直接連絡を取り、著者のためのガイドラインのページに記載されている情報が、雑誌の方針と対応策を正確に反映しているかどうかを確認した。その結果, 学術誌や出版社は、言語の障壁を認識したり軽減したりするためにほとんど取り組みを行っていないことが明らかになった。我々の予想に反して, 学会に属する雑誌は、学会に属さない雑誌と比較して、より言語的に包括的な方針を持っていないようであった。これらの雑誌の多くの方針は透明性と明瞭性を欠いており、不確実性を生み出し、避けることのできる原稿のリジェクトを招き、執筆者と雑誌編集者の両方にさらなる時間と労力を必要とさせる可能性がある。本稿では、言語的に公平な方針の例を紹介し, 科学出版に対する障壁を軽減するために学術雑誌が取ることのできる行動を要約した.

## Introduction

The number of publications in high-impact peer-reviewed journals is a widely adopted measure of a scholar's academic productivity. There is a strong incentive for scientists to publish in internationally recognized, highly ranked journals to promote career advancement and improve their livelihoods (Salager-Meyer 2008; Li 2014; López-Navarro et al. 2015). For instance, successful publication records can increase the consideration of a scholar for other accolades, including award eligibility, hiring, promotion, invitations to give seminars or present at conferences, the selection of case studies to feature in textbooks, media coverage, and citation frequency, all of which likely shape collaborative networks and reinforce existing disparities in career and funding opportunities (Harzing and Metz 2012; Salager-Meyer 2014; Nichols et al. 2020; Niles et al. 2020; Rice et al. 2020; Lynch et al. 2021). Several metrics have been developed to quantify the scientific impact of a journal using publication and citation data, including the *Journal Citation Reports* impact factor score (Carpenter et al. 2014) and the SCImago Journal Rank indicator (González-Pereira et al. 2010). These measures are often perceived as indicative of the quality or rigor of the science published in each journal (Callaham 2002), yet these scores are unrelated to the scientific quality of their articles and are only assigned to a limited and nonrandom selection of journals (Seglen 1997; yKurmis 2003; Nichols et al. 2020). For instance, journals that publish in languages other than English are more likely to be given lower impact factors or to not be assigned a score at all (Seglen 1997; Stegemann 2007). The practice of associating scientific excellence with publication in highly ranked, English-language journals has created a linguistic hegemony in scientific publishing, whereby scholars for whom English is an additional language (EAL scholars) face more barriers to achieving the same level of scientific recognition than scholars who speak English as a first language (Flowerdew 1999; Tardy 2004; Ramírez-Castañeda 2020; Amano et al. 2022). This segregation of access to publishing disproportionately excludes communities from the “Global South,” which refers to populations that experience unequal economic and political relations in the global world order due to historical and current global capitalism and colonialism (Mignolo 2011) and often includes communities from the regions of Latin America, Asia, Africa, and Oceania (Dados and Connell 2012). To achieve equity in science, publishers and scientific journals, especially highly ranked ones, must recognize and alleviate linguistic disparities.

Scientific progress and the dissemination of knowledge are hindered by the English dominance of scientific publishing (Kamadjeu 2019). Ideally, scientific knowledge should be accessible to all, with a free exchange of information among scientists from global communities and users of scientific knowledge, including decision-makers and the general public. Yet, papers published in well-regarded journals are only written in English and are thus inaccessible to communities where English is not widely spoken (Amano et al. 2016; Saha et al. 2019). In contrast, papers written by EAL scholars are largely published in local or regional journals in languages other than English (Salager-Meyer 2014), which may not be recognized by some scientific search engines (Amano et al. 2016; Chowdhury et al. 2022). Thus, scientific knowledge is circulated within specific communities that are linguistically and geographically defined (Collyer 2018; Nuñez et al. 2021). Furthermore, many English-language journals do not permit any of their content to be published in another language, either within the same journal or elsewhere (Horton 2000). This is particularly problematic when research is conducted in countries where English is not a primary language because it could limit the communities relevant to the work from accessing local knowledge that may be crucial for making successful policy, conservation, educational, and development decisions (Shanley and López 2009; Li 2014; Salager-Meyer 2014).

Scholars for whom English is an additional language must often choose between publishing in journals perceived as less prestigious to make their work accessible to non-English-speaking communities versus publishing in more prestigious journals for the betterment of their careers (Meneghini and Packer 2007; Salager-Meyer 2008, 2014; Flowerdew and Li 2009; López-Navarro et al. 2015). Moreover, EAL scholars face obstacles beyond writing their work in English, including navigating editorial or reviewer feedback that negatively focuses on the perceived quality of English writing and the high costs of translation or copyediting services (Herrera 1999; Guardiano et al. 2007; Mungra and Webber 2010; Ramírez-Castañeda 2020). Equality in the interchange of ideas, data, and insights can promote scientific progress and novelty and expand our collective understanding of scientific processes (Grégoire et al. 1995; Amano et al. 2016; Konno et al. 2020). Despite these benefits, our current publication practices discount valuable scientific contributions published in languages other than English while simultaneously imposing barriers for EAL scholars to publish in highly ranked, English-language journals. Inequities within science are maintained, in large part, by the concurrent perpetuation of privilege and exclusion in scientific publishing.

As gatekeepers of professional scientific discourse, publishers and journals are well positioned to make meaningful strides toward achieving equity in science and maximizing effective knowledge transfer among producers and users of scientific knowledge (Li and Flowerdew 2007; Corcoran 2019). Most journals have the autonomy to establish their own policies and practices, including descriptions of expectations for the papers they will consider for publication and explanations of the editorial and review process in their author guidelines (McKinley and Rose 2018). Policies that can accommodate EAL scholars and permit them to share their work more broadly include allowing abstracts, main text, and/or supplementary information in a language other than English, authorizing authors to re-publish their articles elsewhere in a non-English language to increase the accessibility of their work, and assessing submitted articles for their scientific merit and not for their writing style, grammar, or English-language use (Amano, Rios Rojas et al. 2021; Steigerwald et al. 2022).

The descriptions of policies and practices on author guideline pages signal the journal's commitment to inclusivity and equity and can encourage or discourage authors from submitting manuscripts. For example, journal submission guidelines often explicitly require that manuscripts contain a “correct” or “good” standard of English and promise rejection of manuscripts written in “poor” or “non-native” English (Burrough-Boenisch 2006; Heng Hartse and Kubota 2014; McKinley and Rose 2018). Indeed, false assumptions about “standard” English writing being indicative of subject mastery, authority, or scientific quality are ideologically rooted in classism, racism, and xenophobia (Canagarajah 1996; Shapiro et al. 2022; Lippi-Green 2004; Horner et al. 2011). Given such critical, strict, and subjective criteria for acceptable scientific writing, EAL scholars may be justifiably weary of language bias in the editorial or reviewer process and may be anxious or fearful about writing in English and submitting to highly ranked journals (Salager-Meyer 2014; Ramírez-Castañeda 2020). Diversifying editorial boards may help combat bias against perceived English literacy (Harzing and Metz 2012); however, publishers and journals should further reflect on the impacts of their policies and consider mechanisms for alleviating the discriminatory, financial, or time constraints faced by EAL scholars. Lastly, journals that are associated with professional scientific societies may have additional impetus and resources to support their members. Indeed, many society journals have been called upon to help their members determine authorship criteria, achieve gender equality, prevent scientific misconduct, provide awards for conference attendance, adhere to publication ethics, and increase global access to scientific knowledge (Caelleigh 2003; Jones 2003; Doyle et al. 2004; Cadwalader et al. 2014; Segarra et al. 2020). We suspect that journals that publish on behalf of scientific societies are generally more inclusive and active in addressing language-based inequities, but this remains to be formally examined.

Here, we surveyed 230 journals in organismal biology with impact factors of 1.5 or greater for linguistically inclusive and equitable practices and policies. We focused on author guideline pages because they act as the interface and primary point of contact between journals and prospective authors. We collected data on (1) the encouragement of submissions from diverse authors, (2) acknowledgement of the English-hegemony in scientific publishing, (3) policies regarding manuscript rejection based on perceived inadequacies of the English language, (4) the existence of bias-conscious reviewer practices, (5) whether translation and editing resources or services are available, both as a free service or at the author's expense, (6) allowance for non-English abstracts or summaries, or translations of the main text in the supplementary materials, and (7) whether they offer license options that would permit authors (or other scholars) to translate their work and publish it elsewhere (i.e., Creative Commons [CC] licenses, which promote the access and re-use of scholarly work). We directly contacted a subset of journals to verify whether the information on their author guidelines page accurately reflects their policies and the accommodations they would make. For each journal, we also recorded their publisher and the ease of finding specific information on the author guideline page. Furthermore, we examined whether journals associated with professional scientific societies have more inclusive policies. Finally, we provide examples of inclusive policies and a list of suggested actions that publishers, journals, and scientists can consider to address language-based inequities in scientific publishing.

## Methods

### Journal selection

To select journals, we examined the 2019 *Journal Citation Reports* (Clarivate Analytics, 2020) for the following categories: biodiversity and conservation, ecology, entomology, evolution, fish, plant science, and zoology, which resulted in 759 journals. For this study, we considered “highly ranked” journals as those with an impact factor of 1.5 or greater, which is an arbitrary threshold that ensured a large remaining sample size of 352 journals. From there, one person (author JB) inspected the aims and scope sections of journal websites, as well as the titles and abstracts of recently published articles, and excluded any journals that did not convey a strong focus on organismal biology. We excluded (i) journals that primarily focused on the application of methodology or phenomena that occur at other biological levels of organization (e.g., cellular, community, and ecosystem), (ii) journals and book series whose contributions were by invitation only, and (iii) two journals published by the American Museum of Natural History, given that only Museum associates are allowed to publish there. This left 230 journals in our dataset (Supplementary Table 1). We note that our goal is to summarize common policies within the field of organismal biology, and the inclusion or exclusion of particular journals due to the subjective assessment of whether they have a sufficient focus on organismal biology is unlikely to change our overall impression of equitable practices in this discipline.

### Data collection from author guidelines pages

For each journal, we identified the publisher and whether it was published on behalf of a professional society (hereafter “society”). We defined a society as a group of scholars with shared professional interests. We initially screened each society to confirm that membership was open to anyone with interest, but this information was difficult to find on some webpages and we decided to designate any journal associated with a society of any kind as a society journal. We surveyed the author guideline pages of each journal in our dataset for language-inclusive policies and practices. It is possible that journals describe relevant policies and practices elsewhere on their websites, but we did not look at any other webpage beyond the guideline page unless it was obviously relevant and a prominent link was provided. To standardize our assessments and minimize subjective bias, data for each variable (summarized in Table 1) were collected by one person (author BNL) from January through May of 2021 by scanning the author guideline pages and searching for relevant keywords to ensure that important information was not missed (see Table 1 for more detail).

**Table 1:** A summary of the data collected from the author guidelines of journals in our dataset, including the categories used for each variable, potential scoring, the keywords used to verify that we had not missed relevant information when reading the guidelines, and a real or hypothetical example of an equitable policy.

Policy or practice	Scoring	Keywords and search criteria	Examples of equitable policies
The presence of a diversity or inclusion statement that welcomes submissions from diverse authors, broadly defined	Yes No	Keywords: “encourage,” “diverse,” “country,” “countries,” and “background”	Real example earning a Yes score: “It is the mission of the Nordic Society Oikos to advance synthesis in ecology by promoting an open and inclusive science that reflects diversity of approaches, taxa, and environments. The NSO recognizes that these aims can only be reached by welcoming submissions from authors of all origins, races, ethnicities, religions, ages, career stages, gender identities, sexes, sexual orientations, disabilities, or any other individual state.” (*Oiko*s, The Nordic Society Oikos)
Acknowledgement of the barriers EAL scholars can face when publishing in English	Yes No	Keyword: “English”	Hypothetical example earning a Yes score: “We acknowledge that EAL scholars face unique obstacles to publishing in English. We have several policies to help alleviate those barriers.” Real example earning a Yes score: “We realize that publication in foreign journals can be a problem for authors whose first language is not English.” (*Ichthyological Exploration of Freshwaters*)
Acknowledgment that reviewers can be biased by perceived mastery of the English language	Yes No	Keywords: “review” and “reviewer” While several journals mentioned reviewer bias in general on their author guideline pages, our focus was on bias related to English language use.	Hypothetical example earning a Yes score: “Surveys and interviews have confirmed that EAL scholars often receive comments from reviewers of submitted manuscripts that are critical of language use and style. Our editors are committed to ensuring that these biases do not influence editorial decisions.”
A policy of not rejecting submitted papers based on perceived mastery of the English language	Yes Ambiguous No mention No	Keywords: “review”, “reject”, “revision”, and “poor” Journals that discussed the importance of “good” English use without explicitly stating language use could be a basis for rejection were scored as ambiguous.	Real example earning a Yes score: “No paper will be rejected for poor language.” (*Nature Ecology & Evolution*)
Links to resources for translation or editing services	Yes No	Keywords: “English”, “translation”, and “editing”	Real example earning a Yes score: “ASM has a ”buddy system" that includes colleagues who have expressed willingness to assist authors with the presentation of their research. If English is not your primary language, you may request a “buddy system” who will volunteer his or her time to assist you." (*Journal of Mammalogy*, American Society of Mammalogists)
Permission to submit an abstract in a language other than English	Yes No mention No	Keywords: “abstract” We also looked to see whether a title or keywords could be published in another language.	Real example earning a Yes score: “We encourage authors to provide a second abstract in their native language or the language relevant to the country in which the research was conducted.” (*Journal of Ecology*, British Ecological Society)
Permission to submit content in the Supplemental Information in a language other than English	Yes No mention No	Keyword: “supplement”	Hypothetical example earning a Yes score: “To increase accessibility and permit others to cite your work more broadly, authors may include a portion or all of their manuscript in any language as Supplemental Information.”
Publishing model	Open access Closed access Hybrid	We searched broadly on the journal and publisher websites for the types of licenses they offered. Journals were scored as having an open access option if they offered CC licenses (i.e., Gold open access, which allows re-use of the work).	–
The types of CC licenses offered	CC BY CC BY_ND CC BY_SA CC BY_NC CC BY_NC_SA CC BY_NC_ND	We searched broadly on the journal and publisher websites for the types of licenses they offered. It was often difficult to determine whether open access license options were available to all authors or just to those whose funding sources require it.	–

In addition to policy and guideline information, we collected data on the types of CC licenses offered by each journal. These licenses grant public permission for works to be reused, but different license types carry specific stipulations. CC BY licenses allow people to distribute, rearrange, adapt, and build upon the material in any medium or format, so long as the creators of the original material are given proper credit. This is the most flexible and least restrictive of the CC licenses. A journal was not scored as offering this license to all authors if the description indicated that it was only available when required by funding agencies. This license can be expanded to include one or more additional clauses. For instance, CC BY-ND (Non-derivative) licenses prohibit any remixed, transformed, or expanded versions of the original work from being distributed. According to the U.S. Copyright Office, translations are considered a derivative work (Compendium (Third) § 101.1(A)., 2021). CC BY-SA (Share-Alike) licenses stipulate that people must license any modifications of the original material under an identical license. CC BY-NC (Noncommercial) licenses only allow people to distribute, modify, or build upon material for noncommercial purposes. The license itself describes commercial use ambiguously, and may preclude any use that generates nonmonetary commercial advantages (e.g., prestige). This has led to various interpretations of the noncommercial clause by organizations offering the license and by the courts of various countries, and increases the litigation risk for re-users (Hagedorn et al. 2011). Lastly, CC BY licenses can also combine clauses (e.g., CC BY-NC-SA or CC BY-NC-ND).

### Direct email inquiries to journals

We recognize that a lack of information about an inclusive practice does not necessarily mean that it would not be allowed. To confirm whether abstracts and supplementary materials can be published in a second language, we arbitrarily chose 57 (25%) of the journals from our dataset (34 of which were society journals) and emailed those journals using the email addresses for journal administrators or managing editors listed in the contacts section of journal websites. We asked: (1) *Does your journal allow a title, abstract, and keywords in a second language for each accepted manuscript?* (2) *Does your journal allow authors to submit the manuscript in a different language as the supplemental information?* We recognize that data collection on translations will be incomplete in our survey, as some authors may choose to include translations as supplemental information in data repositories in cases where journals prohibit it.

The legal jargon used to describe copyright and licensing policies can make it difficult for authors to determine how their work can be used in the future. Thus, in our direct emails, we asked for clarification on whether copyright and license agreements permit dual-language publication in multiple journals. We further asked: (3) *Do your license agreements permit authors to publish their manuscripts in another journal afterward (for instance, a non-English journal published in another country)?* (4) *Do your license agreements permit authors to publish previously published manuscripts in your journal (so long as the license agreement of the first journal allows it)?*

## Results

**Fig. 1:**
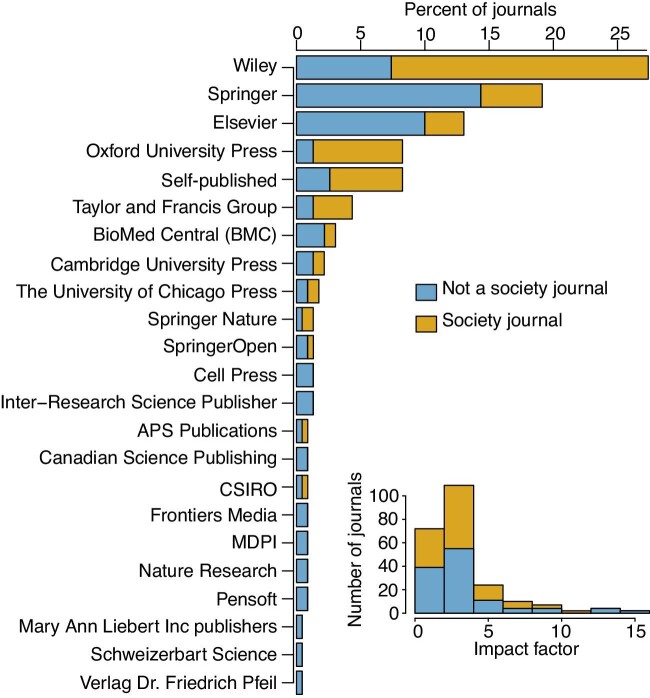
The publishing company, society affiliation status, and impact factors of the journals considered in this study (*n* = 230).

The 230 journals in our study are published by 39 companies (Fig. 1), 72% of which are published by five publishing companies, although we note that this percentage is higher because several of these publishers are not independent (e.g., BioMed Central [BMC] is a part of Springer Nature, Cell Press is an imprint of Elsevier, etc.). Forty-eight percent of journals are published on behalf of professional societies, some of which self-publish. Impact factors range from 1.509 to 14.764, with an overall mean of 3.34 and a median of 2.53. All journals publish exclusively in English except for three, two of which also publish in French and one which will publish in Spanish.

**Fig. 2:**
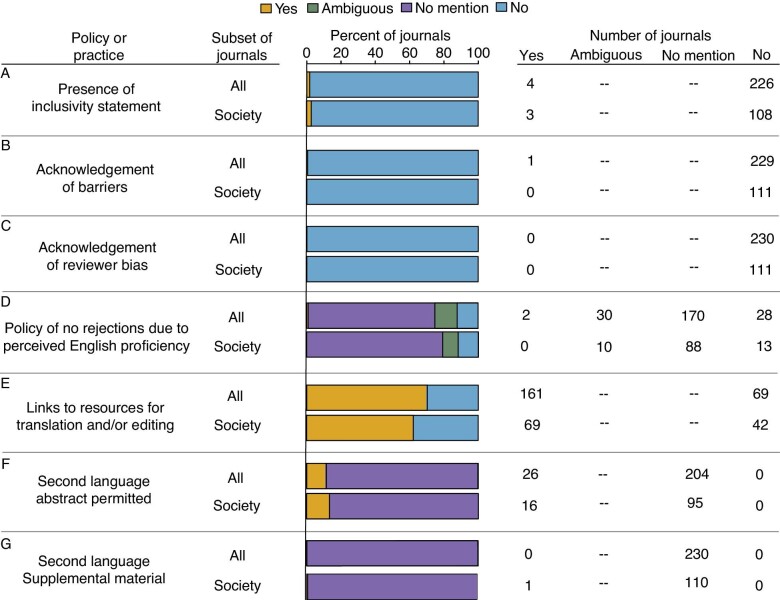
The % and number of all journals (*n* = 230) and of society journals (*n* = 111) with inclusive statements, practices, and policies described in their author guidelines.

Only 1.3% of journals (*n* = 4) had a statement of inclusion at the time of our survey, three of which were society journals (Fig. 2A). Only one journal acknowledged that EAL scholars face additional barriers to publishing or that reviewers can be biased against manuscripts based on English language use (Fig. 2B and C). Only 0.87% of journals (*n* = 2), neither of which was a society journal, explicitly stated that it would not reject a manuscript based on English language quality alone (Fig. 2D). In contrast, 12.1% of journals (*n* = 28, 13 of which are society journals) explicitly stated that manuscripts would be rejected based on perceived English language quality. The adjectives used to describe the criteria for rejection included “*poor*,” “*substandard*,” “*acceptable*,” and “*insufficient*” use of English. One journal recommended authors try to minimize the “annoyance” of their reviewers by having their work reviewed by an English-speaking colleague. Journals that were scored as ambiguously referring to their English-language rejection policy included statements such as “*Editing services can greatly improve the chances of a manuscript being accepted*” and “*You need to ensure the English language is of sufficient quality to be understood*.” The majority of journals did not refer to English language use at all when discussing manuscript rejection.

Links to editing or translation services were provided by 70% of all journals (*n* = 161) and 62.1% of society journals (*n* = 69) spanning 20 publishing companies (Fig. 2E). However, all but two of these journals require payment for their services, and none provided any information of the approximate costs on their author guidelines pages. The two journals that offered complimentary assistance with English editing were society journals. While a limited number of journals permitted abstracts in a language other than English (11.3% of all journals, 14.4% of society journals), the majority of journals did not clarify whether non-English abstracts would be allowed, and only one clarified whether a portion or a summary of the manuscript can be translated in a second language as Supplemental Information (Fig. 2F and G). Of the 11.3% of journals that allowed a second-language abstract (*n* = 26), it was often unclear whether titles and keywords could also be translated, and seven journals seemed to only allow translated abstracts in selected languages (one in Chinese, one in Spanish, two in French, one in Spanish or Portuguese, and two in Spanish, French, German, or Chinese). In addition, one journal required an abstract in Spanish or Portuguese if the presented work had been conducted in Latin America and another required an abstract in Chinese if one or more co-authors were Chinese.

**Fig. 3:**
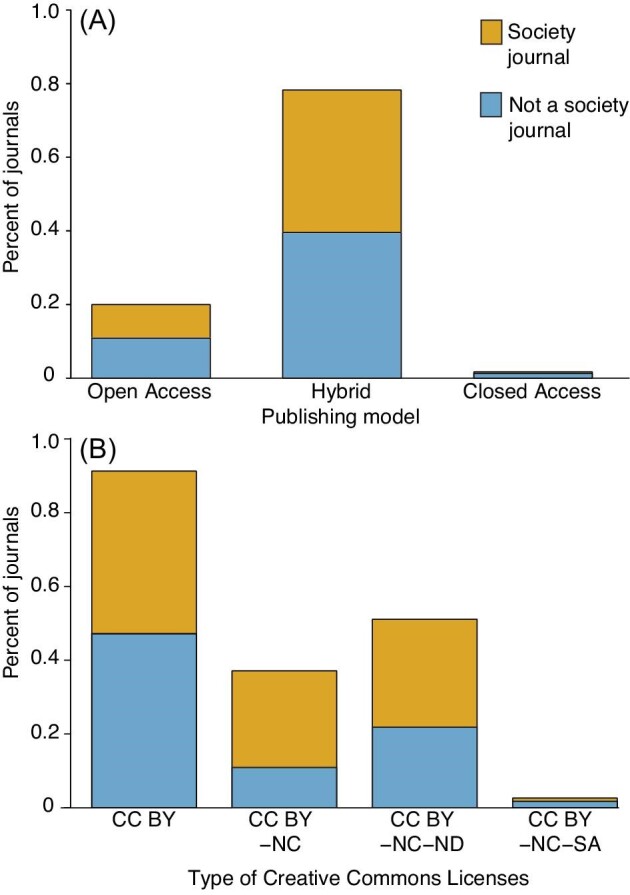
The % of all journals or society journals that (A) have open access, hybrid, or closed access publishing models and (B) offer various Creative Commons license options.

All but four journals had open access or hybrid publishing models (Fig. 3A). The four journals scored as closed access either do not openly or obviously convey their license options or state that CC licenses are only available when funding agencies require it. The most commonly offered CC license is the CC BY license, offered by 90.8% of all journals and 91.0% of society journals, followed by the more restrictive CC BY-NC-ND license (Fig. 3B). We note that although CC BY licenses are broadly offered, many journals have clauses in their license agreements that prohibit authors from submitting translated works that have been published elsewhere, even when the prior license allows it. For instance, SpringerOpen and BMC offer CC BY licenses, however, all of their license agreements require authors to certify that “the article is original” and “has not been formally published in any other peer-reviewed journal” (*License Agreement*, n.d.).

**Fig. 4:**
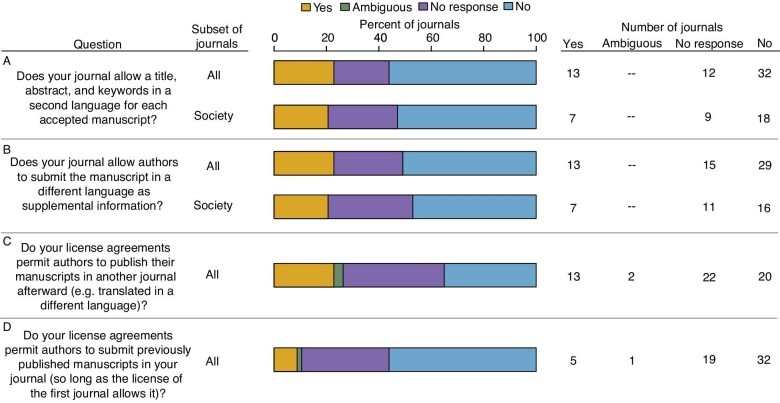
The responses of email inquiries to journals that asked whether second language abstracts and supplemental materials would be permitted, whether manuscripts published in their journal could be later be published in a different language in another journal, and whether they would consider publishing a manuscript that had been previously published in a different language in another journal (should the first license permit it).

Seventy nine percent of journals (*n* = 45 of 57) responded to direct email inquiries about non-English abstracts, supplemental materials, and copyright and license policies, 25 of which were society journals. Respondents often did not answer all questions, so response rates vary by question. Of the 78% of journals (*n* = 45) that responded to the question about second-language abstracts, two confirmed that they would allow it despite not mentioning this possibility on their author guideline pages, suggesting that some journals are more accommodating than their formal policies convey (Fig. 4A). Similarly, of the 42 journals that responded to the inquiry about second-language supplementary material, 13 confirmed they would allow it despite making no mention of it on their author guideline pages (Fig. 4B). This discrepancy is striking. None of the journals we surveyed conveyed this possibility on their author guidelines page, yet 22.8% of all journals and 28% of society journals confirmed it as feasible in direct email inquiries. We note that two journals scored as not permitting second-language content in the Supplemental Information did say they would “consider it under exceptional circumstances” or if “authors could convince the editorial team that it was worthwhile.” In addition, direct email inquiries to journals about translated publications revealed that many editors are unfamiliar that they offer CC BY licenses or what these licenses permit (Fig. 4C and D). A small number of respondents also indicated that they would consider a manuscript for publication that had been previously published in another journal, should the first license permit it, although these accommodations were not reflected on the journal websites.

We note that several publishers have policies that would permit translations of published work even if it was published under a closed license. For instance, all Wiley-owned journals (John Wiley & Sons, Inc.) accept Copyright Transfer Agreements to allow an article previously published in another language to be considered for publication in a Wiley journal. Their website gives this example scenario: “*You have published an article in Spanish. You have translated the article yourself or by employing a translator and want to submit the English translation to a Wiley journal for re-publication. In order to make the translation, you must first approach the Spanish journal for permission to translate the article for republication. The translation itself would then be held under separate copyright by you or the translator. You would then be able to sign the English journal's Copyright Transfer Agreement for the translation, rather than the original article. The translation must include a full bibliographic reference to the original publication, and you must have obtained permission from the original copyright holder to make the translation* (as of April 1, 2021, (*Licensing Info & FAQs | Wiley*, n.d.).”

Similarly, the Oxford University Press (OUP) copyright policy states that “*OUP encourages, for full article reuses only, the translation of its content into other languages”* (*Frequently Asked Questions OUP*, n.d.). BMC policy states that *“Secondary publication of material published in other journals or online may be justifiable and beneficial, especially when intended to disseminate important information to the widest possible audience”* and *“Authors should … seek approval from the original publisher to check that they do not breach the copyright terms of the original publication and that the original publisher gives permission for publication of the translation under the Creative Commons Attribution License 4.0”* (*ICMJE | Recommendations | Overlapping Publications*, n.d.). Finding these policies requires searching carefully on various journal and publisher web pages, as they were generally not referred to on the author guidelines pages. However, it seems that translations of scholarly work could occur more frequently than they currently do via these mechanisms if they were communicated more prominently.

## Discussion

Our survey revealed that highly ranked journals in organismal biology have made little progress toward promoting linguistic equity. Counter to our predictions, journals associated with scientific societies did not appear to have more inclusive policies compared to non-society journals. Exclusionary policies impose additional barriers for EAL scholars, and policies that lack transparency and clarity are similarly damaging as they generate uncertainty, result in avoidable manuscript rejections, and necessitate additional time and effort from both EAL scholars and journal editors. The current publishing culture and practices prohibit equal participation in science, which can negatively impact the career trajectory of EAL scholars (Salager-Meyer 2008; Li 2014; López-Navarro et al. 2015) and limit scientific progress and innovation (Urbina-Blanco et al. 2020). In cases where publishers dictate major policy decisions for the journals they manage, we suggest individual journals collaborate to advocate for change and to consider switching publishers as an allied group. Below, we feature examples of equitable policies and summarize actions that journals can take to begin to alleviate language barriers to scientific publishing.

**Fig. 5:**
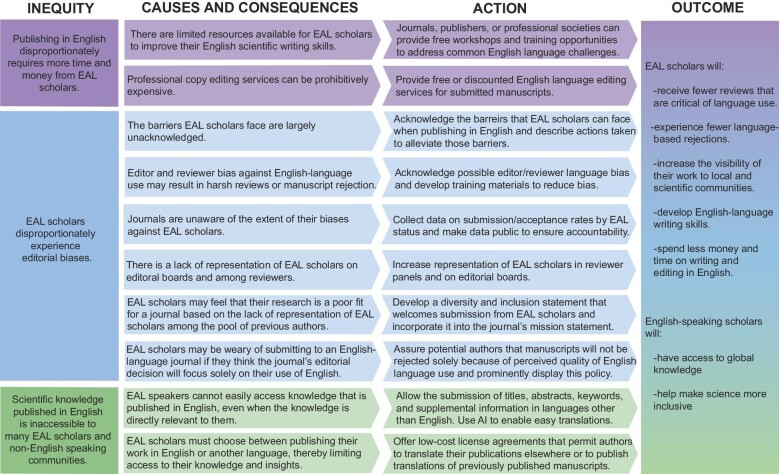
Examples of actions journals and societies can take to begin to overcome language barriers in scientific publishing and their impact on both EAL and English-speaker scholars.

We recommend the editorial team of each journal expand their mission and values statements to specifically address and value the scientist as well as the science (Fig. 5). A mission statement that identifies the community of readers and authors that the journal serves could help editors identify inequitable policies that prevent their intended readers and authors from accessing or contributing to the journal. A clear and inclusive mission statement would communicate the journal's values to the broader scientific community and can motivate action toward alleviating barriers to access and opportunity for the journal's intended readers and authors. Journals that desire to serve a more inclusive and global community should develop a strategic plan to assess the equity of their policies, address any shortcomings, and transparently communicate their progress.

In the short term, journals could audit their author guidelines to clarify their policies and practices and positively impact the scientific publication process for EAL scholars (Fig. 5). We suggest that journals need to clarify whether translations of various components of a manuscript are allowed, highlight whom potential authors can contact with questions, prominently and directly explain license and copyright policies, educate editors and anyone contacted by potential authors on copyright policies, clearly indicate the availability of translated versions of articles on their websites, and acknowledge translators and translation platforms. To determine the extent of inequities that exist, journals can quantify publication biases against EAL scholars by collecting author demographic data, integrating them in examinations of submission and acceptance metrics, and publishing this information on their websites to enhance transparency and accountability (Nuñez et al. 2019). Similar attention to the demographic composition of editors and reviewers may further reduce biases (e.g., Elsevier journals show the gender composition of editors: https://www.sciencedirect.com/journal/biological-conservation/about/editorial-board). Editors can familiarize themselves with data-driven research on language barriers to inform effective actions to alleviate inequities. Potential preliminary actions include setting achievable goals based on demographic and publication data and regularly assessing progress toward goals. In addition to providing options for commercial English editing services, journals can also develop opportunities and programs for EAL scholars to develop their English-language writing skills. For instance, journals could work with their associate editors to identify the most common areas for improvement of English-language use in submitted manuscripts and offer online workshops and resources for addressing them. Lastly, journals, publishers, and indexing platforms (e.g., Web of Science, Google Scholar, PubMed, etc.) might consider using machine translation systems to enable quick and affordable translations (Steigerwald et al. 2022) after assessing their accuracy and quality (Amano, Berdejo-Espinola et al. 2021).

Several society journals provide free assistance with English-language use and grammar to enhance the professional development of their members, alleviate financial or linguistic barriers to publication, or increase the global sharing of scientific knowledge. For instance, the American Society of Mammalogists has formed a “buddy system” through which EAL authors who submit a manuscript to the *Journal of Mammalogy* can be matched with an English-speaking member of the society for free assistance with copyediting prior to the manuscript going for review (Álvarez-Castañeda et al. 2019). The Association of Field Ornithologists’ Editorial Assistance Program offers an analogous service for submissions to the *Journal of Field Ornithology*, as does the The Wildlife Society for *The Journal of Wildlife Management*. The Society for Conservation Biologists’ Publication Partners Program will offer authors the option of additional input from experienced scientists on manuscripts written in English and submitted to *Conservation Biology*, and they also allow authors to add versions of their manuscripts in other languages as supporting information (Burgman et al. 2015). At the time of this study, the Partners Program was not described on the author guidelines page of *Conservation Biology*, although there was a link to it in one of the menus. We acknowledge that programs like these take time and effort to implement. Indeed, addressing inequities and alleviating barriers of any kind requires sacrifices and dedication from those with privilege. However, in the case of language assistance programs, a large volunteer network (i.e., composed of society members or past authors of the journal) can reduce the commitment needed from individual language editors.

In addition to equitable guidelines and policies, journals can adopt publishing models that maximize the access and impact of scientific knowledge. For instance, ideally, all manuscripts would be published open access and with a license that permits translation. We found that many journals offer options that permit translations to their authors as part of their open access initiatives. However, publishing under flexible licenses, like the CC BY license, often costs more than publishing under a closed or partially open access license. We would be remiss not to emphasize that the major fallacy of the open access model is that it relies on an author-pays business model meant to mitigate article processing charges (Hagenhoff et al. 2008; Istratii et al. 2020). These costs can be prohibitive to scholars in the Global South, where institutions and funding sources rarely fund article processing fees (Mekonnen et al. 2021). Thus, open access publishing models can further impede academic publishing for EAL scholars, as has already been observed in the discipline of ecology, where the rate of manuscript submissions is lower for scholars from both the Global South and countries where English is not widely spoken (Nuñez et al. 2019). Indeed, the geographic diversity of authors was lower in open access journals than in hybrid journals included in Elsevier's “Mirror journal” system, in which pairs of hybrid and open access journals share editorial boards and standards for acceptance (Smith et al. 2021). A 2012 study surveyed 429 researchers in 65 countries across 7 different disciplines about the article processing charges for their recent publications (Solomon and Björk 2012). When categorizing the researchers based on Gross National Product (GNP) of >${\$}$25,000 or <${\$}$25,000 (USD), 39% of authors from countries under ${\$}$25,000 GNP paid with personal funds, whereas only 11% of authors from wealthier countries used personal funds. Therefore, the current open-access model reinforces epistemological inequalities through global wealth disparities such that authors from the wealthier countries of the Global North have the necessary institutional capital to pay for open access fees while authors from the Global South often have to use personal funds. In fact, many governmental agencies in the Global North strongly encourage or require all nationally funded research to be published open access. For example, in 2018, Science Europe launched Plan S, which dictates that all publicly funded research must be published with open access, regardless of institutional funding to do so, as “all scientists should be able to publish their work with open access even if their institutions have limited means” (Haug 2019). The exclusionary costs of open access publication can be offset by offering lower fees or waivers to scholars from the Global South or to those who otherwise demonstrate need. To further reduce language barriers, societies may consider switching from for-profit to nonprofit publishers, and privileged authors can likewise decide to publish in journals with equitable policies.

We recognize that our recommendations reflect preliminary and relatively low-impact steps toward reducing barriers to publication for EAL scholars and that they do not address the larger, prevailing issues that perpetuate disparities in access and opportunities. To achieve equity in scientific publishing, we must reject pay-to-publish models in favor of author-led, nonprofit models (Debat and Babini 2020). Additionally, institutions and funding agencies must broaden the kinds of scholarship they consider indicative of scientific productivity and merit, which should include celebrating diversity in linguistic choices (Canagarajah 2022) and valuing translations of publications in multiple journals and in venues that disseminate knowledge to the general public. Abandoning the status quo in scientific publishing requires an enormous cultural shift at all levels of the global scientific community, including individual scientists, journals, publishers, departments, institutions, and funding agencies. The responsibility for catalyzing this cultural revolution largely falls on those with privilege, and we call on scientific communities and institutional leaders to initiate and promote innovative, equitable, and impactful change.

## Supplementary Material

obad003_Supplemental_Files
